# Supplementary material for: Coping with Trauma and Symptoms of Post-Traumatic Stress Disorder: Exploring Intentions and Lay Beliefs about Appropriate Strategies among Asylum-Seeking Migrants from Sub-Saharan Africa in Germany
Source: Int J Environ Res Public Health. 2022 Feb 4;19(3):1783. doi: 10.3390/ijerph19031783 (PMC8834703; doi:10.3390/ijerph19031783)
Supplement: Supplementary file 1 [file ijerph-19-01783-s001.zip › Supplementary material_vignette.pdf]

**Table S1.** The vignette adapted from (Maercker, 2013).

| |
|---|
| "Since the [flight/armed robbery], I have become a totally different person. In the evenings, I lie in bed and then these thoughts and images come and I lie awake forever. Now I have reached a point where I realize I can't go on like this anymore… Sometimes I scream at night and I wake up drenched in sweat because of the nightmares. If I have arrived somewhere and there is a noise, I wince. There it is again. I can't turn it off, it's like an electric shock that immediately goes straight up and triggers intense sweating. My wife/My husband accuses me of often being aggressive, easily irritable and she/he is afraid of my outbursts of rage. That's why I prefer to withdraw myself because I always have a feeling that no one can be trusted anymore. Many things just don't interest me anymore. Sometimes my environment appears distant and unreal and I have a feeling of "standing next to myself", then I become totally numb. Afterwards I sometimes can't remember what has happened. I have no hope left anymore…" |
